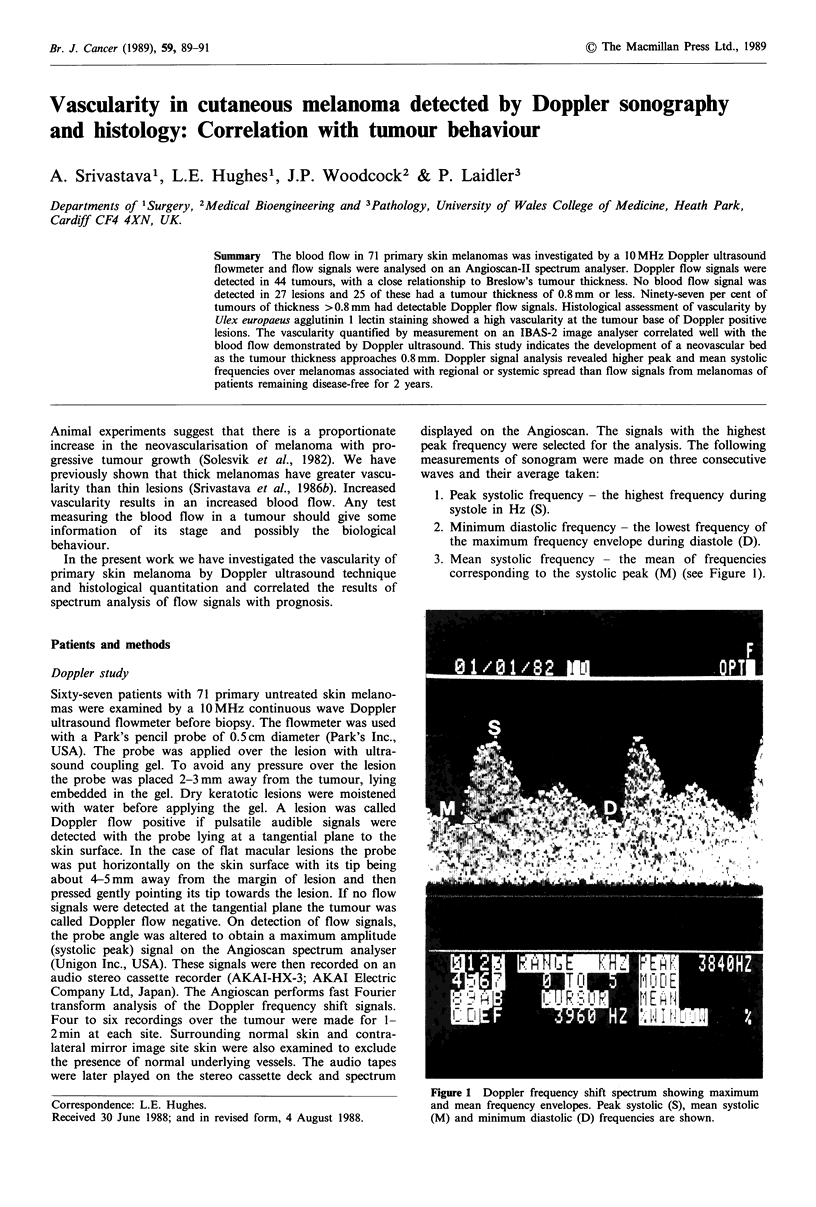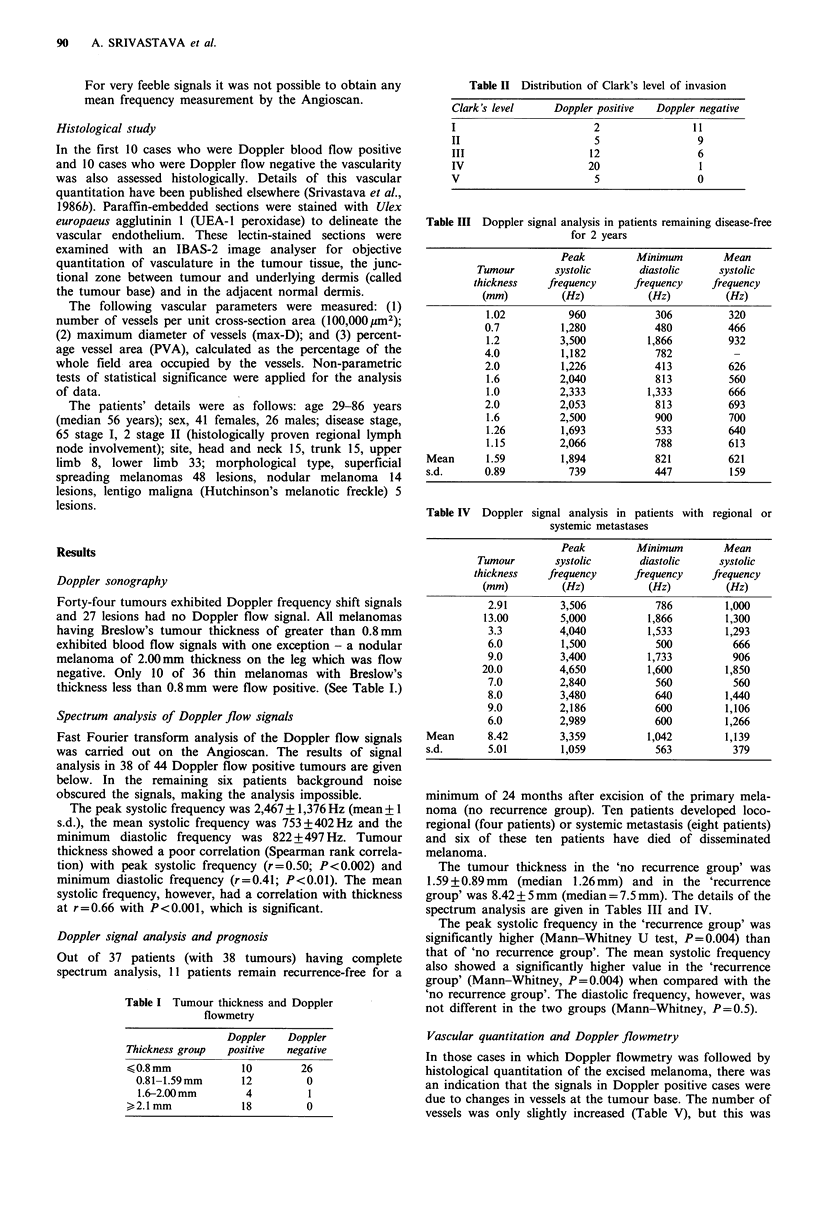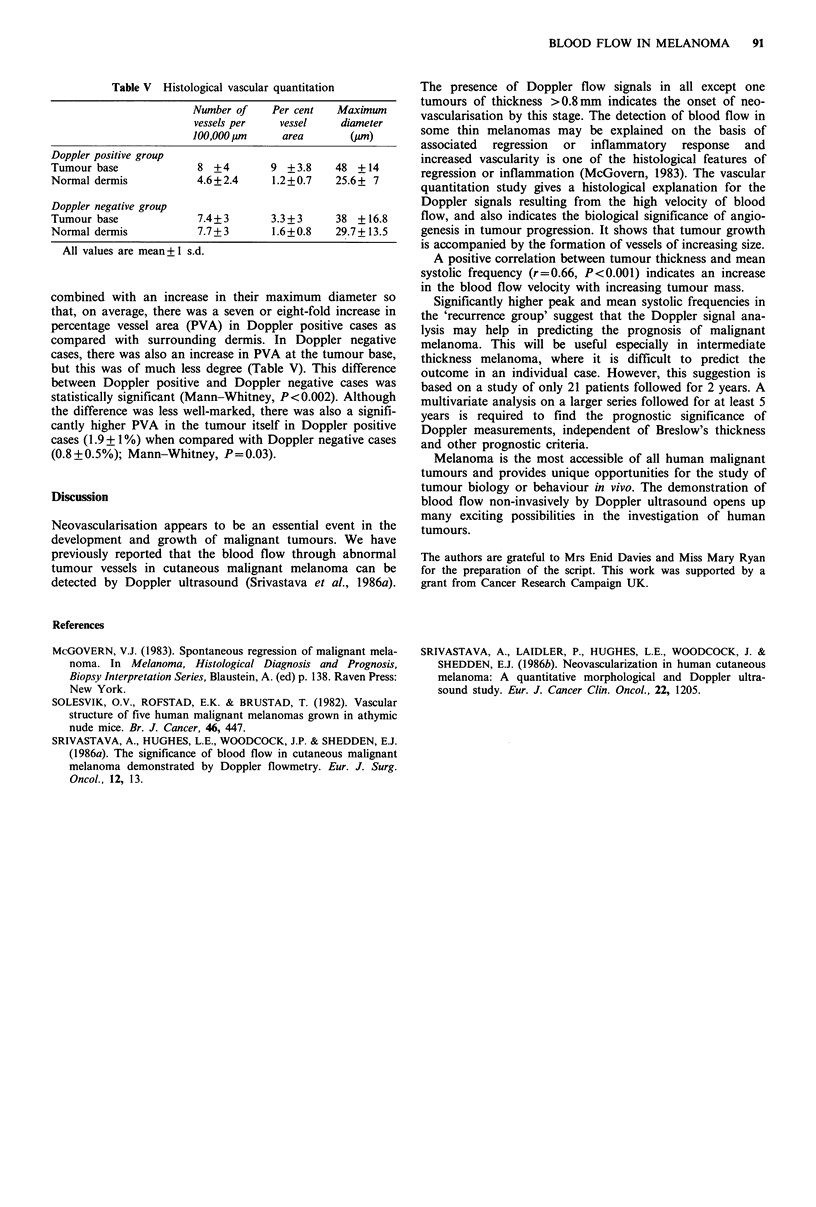# Vascularity in cutaneous melanoma detected by Doppler sonography and histology: correlation with tumour behaviour.

**DOI:** 10.1038/bjc.1989.17

**Published:** 1989-01

**Authors:** A. Srivastava, L. E. Hughes, J. P. Woodcock, P. Laidler

**Affiliations:** Department of Surgery, University of Wales College of Medicine, Heath Park, Cardiff, UK.

## Abstract

**Images:**


*Br. J. Cancer* (1989), **59**, 89–91 © The Macmillan Press Ltd., 1989

### Vascularity in cutaneous melanoma detected by Doppler sonography and histology: Correlation with tumour behaviour

A. Srivastava[1], L.E. Hughes[1], J.P. Woodcock[2] & P. Laidler[3]

*Departments of [1]Surgery, [2]Medical Bioengineering and [3]Pathology, University of Wales College of Medicine, Heath Park, Cardiff CF4 4XN, UK.*

**Summary** The blood flow in 71 primary skin melanomas was investigated by a 10 MHz Doppler ultrasound flowmeter and flow signals were analysed on an Angioscan-II spectrum analyser. Doppler flow signals were detected in 44 tumours, with a close relationship to Breslow's tumour thickness. No blood flow signal was detected in 27 lesions and 25 of these had a tumour thickness of 0.8 mm or less. Ninety-seven per cent of tumours of thickness >0.8 mm had detectable Doppler flow signals. Histological assessment of vascularity by *Ulex europaeus* agglutinin 1 lectin staining showed a high vascularity at the tumour base of Doppler positive lesions. The vascularity quantified by measurement on an IBAS-2 image analyser correlated well with the blood flow demonstrated by Doppler ultrasound. This study indicates the development of a neovascular bed as the tumour thickness approaches 0.8 mm. Doppler signal analysis revealed higher peak and mean systolic frequencies over melanomas associated with regional or systemic spread than flow signals from melanomas of patients remaining disease-free for 2 years.

Animal experiments suggest that there is a proportionate increase in the neovascularisation of melanoma with progressive tumour growth (Solesvik *et al.*, 1982). We have previously shown that thick melanomas have greater vascularity than thin lesions (Srivastava *et al.*, 1986*b*). Increased vascularity results in an increased blood flow. Any test measuring the blood flow in a tumour should give some information of its stage and possibly the biological behaviour.

In the present work we have investigated the vascularity of primary skin melanoma by Doppler ultrasound technique and histological quantitation and correlated the results of spectrum analysis of flow signals with prognosis.

### Patients and methods

### *Doppler study*

Sixty-seven patients with 71 primary untreated skin melanomas were examined by a 10 MHz continuous wave Doppler ultrasound flowmeter before biopsy. The flowmeter was used with a Park's pencil probe of 0.5 cm diameter (Park's Inc., USA). The probe was applied over the lesion with ultrasound coupling gel. To avoid any pressure over the lesion the probe was placed 2–3 mm away from the tumour, lying embedded in the gel. Dry keratotic lesions were moistened with water before applying the gel. A lesion was called Doppler flow positive if pulsatile audible signals were detected with the probe lying at a tangential plane to the skin surface. In the case of flat macular lesions the probe was put horizontally on the skin surface with its tip being about 4–5 mm away from the margin of lesion and then pressed gently pointing its tip towards the lesion. If no flow signals were detected at the tangential plane the tumour was called Doppler flow negative. On detection of flow signals, the probe angle was altered to obtain a maximum amplitude (systolic peak) signal on the Angioscan spectrum analyser (Unigon Inc., USA). These signals were then recorded on an audio stereo cassette recorder (AKAI-HX-3; AKAI Electric Company Ltd, Japan). The Angioscan performs fast Fourier transform analysis of the Doppler frequency shift signals. Four to six recordings over the tumour were made for 1–2 min at each site. Surrounding normal skin and contralateral mirror image site skin were also examined to exclude the presence of normal underlying vessels. The audio tapes were later played on the stereo cassette deck and spectrum displayed on the Angioscan. The signals with the highest peak frequency were selected for the analysis. The following measurements of sonogram were made on three consecutive waves and their average taken:

1. Peak systolic frequency – the highest frequency during systole in Hz (S).
2. Minimum diastolic frequency – the lowest frequency of the maximum frequency envelope during diastole (D).
3. Mean systolic frequency – the mean of frequencies corresponding to the systolic peak (M) (see Figure 1).

**Figure 1** Doppler frequency shift spectrum showing maximum and mean frequency envelopes. Peak systolic (S), mean systolic (M) and minimum diastolic (D) frequencies are shown.

Correspondence: L.E. Hughes.
Received 30 June 1988; and in revised form, 4 August 1988.

For very feeble signals it was not possible to obtain any mean frequency measurement by the Angioscan.

*Histological study*

In the first 10 cases who were Doppler blood flow positive and 10 cases who were Doppler flow negative the vascularity was also assessed histologically. Details of this vascular quantitation have been published elsewhere (Srivastava *et al.*, 1986*b*). Paraffin-embedded sections were stained with *Ulex europaeus* agglutinin 1 (UEA-1 peroxidase) to delineate the vascular endothelium. These lectin-stained sections were examined with an IBAS-2 image analyser for objective quantitation of vasculature in the tumour tissue, the junctional zone between tumour and underlying dermis (called the tumour base) and in the adjacent normal dermis.

The following vascular parameters were measured: (1) number of vessels per unit cross-section area (100,000 $\mu m^2$); (2) maximum diameter of vessels (max-D); and (3) percentage vessel area (PVA), calculated as the percentage of the whole field area occupied by the vessels. Non-parametric tests of statistical significance were applied for the analysis of data.

The patients' details were as follows: age 29–86 years (median 56 years); sex, 41 females, 26 males; disease stage, 65 stage I, 2 stage II (histologically proven regional lymph node involvement); site, head and neck 15, trunk 15, upper limb 8, lower limb 33; morphological type, superficial spreading melanomas 48 lesions, nodular melanoma 14 lesions, lentigo maligna (Hutchinson's melanotic freckle) 5 lesions.

### Results

*Doppler sonography*

Forty-four tumours exhibited Doppler frequency shift signals and 27 lesions had no Doppler flow signal. All melanomas having Breslow's tumour thickness of greater than 0.8 mm exhibited blood flow signals with one exception – a nodular melanoma of 2.00 mm thickness on the leg which was flow negative. Only 10 of 36 thin melanomas with Breslow's thickness less than 0.8 mm were flow positive. (See Table I.)

*Spectrum analysis of Doppler flow signals*

Fast Fourier transform analysis of the Doppler flow signals was carried out on the Angioscan. The results of signal analysis in 38 of 44 Doppler flow positive tumours are given below. In the remaining six patients background noise obscured the signals, making the analysis impossible.

The peak systolic frequency was 2,467 ± 1,376 Hz (mean ± 1 s.d.), the mean systolic frequency was 753 ± 402 Hz and the minimum diastolic frequency was 822 ± 497 Hz. Tumour thickness showed a poor correlation (Spearman rank correlation) with peak systolic frequency ($r = 0.50$; $P < 0.002$) and minimum diastolic frequency ($r = 0.41$; $P < 0.01$). The mean systolic frequency, however, had a correlation with thickness at $r = 0.66$ with $P < 0.001$, which is significant.

*Doppler signal analysis and prognosis*

Out of 37 patients (with 38 tumours) having complete spectrum analysis, 11 patients remain recurrence-free for a minimum of 24 months after excision of the primary melanoma (no recurrence group). Ten patients developed loco-regional (four patients) or systemic metastasis (eight patients) and six of these ten patients have died of disseminated melanoma.

**Table I** Tumour thickness and Doppler flowmetry

| *Thickness group* | *Doppler positive* | *Doppler negative* |
|---|---|---|
| ≤0.8 mm | 10 | 26 |
| 0.81–1.59 mm | 12 | 0 |
| 1.6–2.00 mm | 4 | 1 |
| ≥2.1 mm | 18 | 0 |

**Table II** Distribution of Clark's level of invasion

| *Clark's level* | *Doppler positive* | *Doppler negative* |
|---|---|---|
| I | 2 | 11 |
| II | 5 | 9 |
| III | 12 | 6 |
| IV | 20 | 1 |
| V | 5 | 0 |

**Table III** Doppler signal analysis in patients remaining disease-free for 2 years

| | *Tumour thickness (mm)* | *Peak systolic frequency (Hz)* | *Minimum diastolic frequency (Hz)* | *Mean systolic frequency (Hz)* |
|---|---|---|---|---|
| | 1.02 | 960 | 306 | 320 |
| | 0.7 | 1,280 | 480 | 466 |
| | 1.2 | 3,500 | 1,866 | 932 |
| | 4.0 | 1,182 | 782 | – |
| | 2.0 | 1,226 | 413 | 626 |
| | 1.6 | 2,040 | 813 | 560 |
| | 1.0 | 2,333 | 1,333 | 666 |
| | 2.0 | 2,053 | 813 | 693 |
| | 1.6 | 2,500 | 900 | 700 |
| | 1.26 | 1,693 | 533 | 640 |
| | 1.15 | 2,066 | 788 | 613 |
| Mean | 1.59 | 1,894 | 821 | 621 |
| s.d. | 0.89 | 739 | 447 | 159 |

**Table IV** Doppler signal analysis in patients with regional or systemic metastases

| | *Tumour thickness (mm)* | *Peak systolic frequency (Hz)* | *Minimum diastolic frequency (Hz)* | *Mean systolic frequency (Hz)* |
|---|---|---|---|---|
| | 2.91 | 3,506 | 786 | 1,000 |
| | 13.00 | 5,000 | 1,866 | 1,300 |
| | 3.3 | 4,040 | 1,533 | 1,293 |
| | 6.0 | 1,500 | 500 | 666 |
| | 9.0 | 3,400 | 1,733 | 906 |
| | 20.0 | 4,650 | 1,600 | 1,850 |
| | 7.0 | 2,840 | 560 | 560 |
| | 8.0 | 3,480 | 640 | 1,440 |
| | 9.0 | 2,186 | 600 | 1,106 |
| | 6.0 | 2,989 | 600 | 1,266 |
| Mean | 8.42 | 3,359 | 1,042 | 1,139 |
| s.d. | 5.01 | 1,059 | 563 | 379 |

The tumour thickness in the 'no recurrence group' was 1.59 ± 0.89 mm (median 1.26 mm) and in the 'recurrence group' was 8.42 ± 5 mm (median = 7.5 mm). The details of the spectrum analysis are given in Tables III and IV.

The peak systolic frequency in the 'recurrence group' was significantly higher (Mann–Whitney U test, $P = 0.004$) than that of 'no recurrence group'. The mean systolic frequency also showed a significantly higher value in the 'recurrence group' (Mann–Whitney, $P = 0.004$) when compared with the 'no recurrence group'. The diastolic frequency, however, was not different in the two groups (Mann–Whitney, $P = 0.5$).

*Vascular quantitation and Doppler flowmetry*

In those cases in which Doppler flowmetry was followed by histological quantitation of the excised melanoma, there was an indication that the signals in Doppler positive cases were due to changes in vessels at the tumour base. The number of vessels was only slightly increased (Table V), but this was

**Table V** Histological vascular quantitation

| | *Number of vessels per 100,000 μm* | *Per cent vessel area* | *Maximum diameter (μm)* |
|---|---|---|---|
| *Doppler positive group* | | | |
| Tumour base | 8 ±4 | 9 ±3.8 | 48 ±14 |
| Normal dermis | 4.6±2.4 | 1.2±0.7 | 25.6± 7 |
| *Doppler negative group* | | | |
| Tumour base | 7.4±3 | 3.3±3 | 38 ±16.8 |
| Normal dermis | 7.7±3 | 1.6±0.8 | 29.7±13.5 |

All values are mean±1 s.d.

combined with an increase in their maximum diameter so that, on average, there was a seven or eight-fold increase in percentage vessel area (PVA) in Doppler positive cases as compared with surrounding dermis. In Doppler negative cases, there was also an increase in PVA at the tumour base, but this was of much less degree (Table V). This difference between Doppler positive and Doppler negative cases was statistically significant (Mann–Whitney, $P<0.002$). Although the difference was less well-marked, there was also a significantly higher PVA in the tumour itself in Doppler positive cases (1.9±1%) when compared with Doppler negative cases (0.8±0.5%); Mann–Whitney, $P=0.03$).

### Discussion

Neovascularisation appears to be an essential event in the development and growth of malignant tumours. We have previously reported that the blood flow through abnormal tumour vessels in cutaneous malignant melanoma can be detected by Doppler ultrasound (Srivastava *et al.*, 1986*a*). The presence of Doppler flow signals in all except one tumours of thickness >0.8 mm indicates the onset of neovascularisation by this stage. The detection of blood flow in some thin melanomas may be explained on the basis of associated regression or inflammatory response and increased vascularity is one of the histological features of regression or inflammation (McGovern, 1983). The vascular quantitation study gives a histological explanation for the Doppler signals resulting from the high velocity of blood flow, and also indicates the biological significance of angiogenesis in tumour progression. It shows that tumour growth is accompanied by the formation of vessels of increasing size.

A positive correlation between tumour thickness and mean systolic frequency ($r=0.66$, $P<0.001$) indicates an increase in the blood flow velocity with increasing tumour mass.

Significantly higher peak and mean systolic frequencies in the 'recurrence group' suggest that the Doppler signal analysis may help in predicting the prognosis of malignant melanoma. This will be useful especially in intermediate thickness melanoma, where it is difficult to predict the outcome in an individual case. However, this suggestion is based on a study of only 21 patients followed for 2 years. A multivariate analysis on a larger series followed for at least 5 years is required to find the prognostic significance of Doppler measurements, independent of Breslow's thickness and other prognostic criteria.

Melanoma is the most accessible of all human malignant tumours and provides unique opportunities for the study of tumour biology or behaviour *in vivo*. The demonstration of blood flow non-invasively by Doppler ultrasound opens up many exciting possibilities in the investigation of human tumours.

The authors are grateful to Mrs Enid Davies and Miss Mary Ryan for the preparation of the script. This work was supported by a grant from Cancer Research Campaign UK.

### References

McGOVERN, V.J. (1983). Spontaneous regression of malignant melanoma. In *Melanoma, Histological Diagnosis and Prognosis, Biopsy Interpretation Series*, Blaustein, A. (ed) p. 138. Raven Press: New York.

SOLESVIK, O.V., ROFSTAD, E.K. & BRUSTAD, T. (1982). Vascular structure of five human malignant melanomas grown in athymic nude mice. *Br. J. Cancer*, **46**, 447.

SRIVASTAVA, A., HUGHES, L.E., WOODCOCK, J.P. & SHEDDEN, E.J. (1986*a*). The significance of blood flow in cutaneous malignant melanoma demonstrated by Doppler flowmetry. *Eur. J. Surg. Oncol.*, **12**, 13.

SRIVASTAVA, A., LAIDLER, P., HUGHES, L.E., WOODCOCK, J. & SHEDDEN, E.J. (1986*b*). Neovascularization in human cutaneous melanoma: A quantitative morphological and Doppler ultrasound study. *Eur. J. Cancer Clin. Oncol.*, **22**, 1205.